# Supplementary material for: Clinical benefit of cancer drugs approved in Switzerland 2010–2019
Source: PLoS One. 2022 Jun 10;17(6):e0268545. doi: 10.1371/journal.pone.0268545 (PMC9187080; doi:10.1371/journal.pone.0268545)
Supplement: S1 Table — (DOCX) [file pone.0268545.s001.docx]

**Supplementary S1 Table**: Grades evaluated with the ESMO-MCBS v1.1, the ASCO-VF v2 and the OLUtool v2.

|  | | | | | |  |
| --- | --- | --- | --- | --- | --- | --- |
|  |  |  |  |  |  |  |
|  | | | | | |  |
| Clinical Benefit | Grade | *N* (%) |  | Grade | *N* (%) |  |
|  | **ESMO-MCBS v1.1** | | | | |  |
|  | Curative (*N*=7) | |  | Palliative (*N*=93) | |  |
| High | A | 5 (71) |  | 5 | 4 (4) |  |
|  |  |  |  | 4 | 29 (31) |  |
|  | B | 1 (14) |  | 3 | 37 (40) |  |
|  |  |  |  | 2 | 15 (16) |  |
| Low | C | 1 (14) |  | 1 | 8 (9) |  |
|  | **ASCO-VF v2** | | | | |  |
|  | Curative (*N*=6) | |  | Palliative (*N*=80) | |  |
| High | ≥45p | 1 (17) |  |  | 43 (54) |  |
| Low | <45p | 5 (83) |  |  | 37 (46) |  |
|  | **OLUtool v2** | | | | |  |
|  | Curative (*N*=6) | |  | Palliative (*N*=91) | |  |
| High | A | 2 (33) |  | A | 19 (21) |  |
|  | B | 2 (33) |  | B | 22 (24) |  |
|  | C | 1 (17) |  | C | 34 (37) |  |
| Low | D | 1 (17) |  | D | 16 (18) |  |
| Abbreviations: ESMO-MCBS v1.1: European Society for Medical Oncology - Magnitude of Clinical Benefit Scale version 1.1; ASCO-VF v2: American Society of Clinical Oncology - Value Framework version 2; OLUtool v2: OLUtool version 2; *N*: number; p: points | | | | | |  |
