# Supplementary material for: Clinical benefit of cancer drugs approved in Switzerland 2010–2019
Source: PLoS One. 2022 Jun 10;17(6):e0268545. doi: 10.1371/journal.pone.0268545 (PMC9187080; doi:10.1371/journal.pone.0268545)
Supplement: S2 Table — (DOCX) [file pone.0268545.s002.docx]

**Supplementary S2 Table**. Sensitivity analysis for the correlation and concordance between the studies with the optimal threshold calculated with ROC curve analyses for ASCO-VF v2.

|  |  |  |  |  |  |
| --- | --- | --- | --- | --- | --- |
|  | ESMO-MCBS/ASCO-VF v2 | |  | ASCO-VF v2/OLUtool v2 | |
|  | all studies (*N=*86) | palliative setting (*N=*80) |  | all studies (*N=*84) | palliative setting (*N=*78) |
| Optimal cut-off for ASCO-VF v2 | 46.6 p | 49.15 p |  | 55.65 p | 55.65 p |
| Number of concordant studies | 56 (65%) | 56 (70%) |  | 54 (64%) | 52 (67%) |
| Spearman's rho |  | 0.42 (*P*<0.001) |  |  | 0.40 (*P*<0.001) |
| Cohen's Kappa | 0.30 (*P*=0.005) | 0.39 (*P*<0.001) |  | 0.31 (*P*=0.001) | 0.35 (*P*<0.001) |
| Abbreviations: ESMO-MCBS v1.1: European Society for Medical Oncology - Magnitude of Clinical Benefit Scale version 1.1; ASCO-VF v2: American Society of Clinical Oncology - Value Framework version 2; OLUtool v2: OLUtool version 2; p: points; *P:* p-value; | | | | | |
