# Supplementary material for: Clinical benefit of cancer drugs approved in Switzerland 2010–2019
Source: PLoS One. 2022 Jun 10;17(6):e0268545. doi: 10.1371/journal.pone.0268545 (PMC9187080; doi:10.1371/journal.pone.0268545)
Supplement: S3 Table — (DOCX) [file pone.0268545.s003.docx]

| **Table A.3a. Sensitivity analysis: Concordance and correlation of studies in which the same outcomes were evaluated between the different frameworks** | | | | | | | | | | | | | | | | | | | | | | | | | | |  |
| --- | --- | --- | --- | --- | --- | --- | --- | --- | --- | --- | --- | --- | --- | --- | --- | --- | --- | --- | --- | --- | --- | --- | --- | --- | --- | --- | --- |
|  | ESMO-MCBS/ASCO-VF v2 | | | | |  | | ESMO-MCBS/OLUtool v2 | | | | | | | | | |  | | ASCO-VF v2/OLUtool v2 | | | | | | |  |
|  | all studies (*N=*80) | | palliative setting (*N*=74) | curative setting (*N=*6) | |  | | all studies (*N=*93) | | | | palliative setting (*N=*87) | | curative setting (*N=*6) | | | |  | | all studies (*N=*74) | | | palliative setting (*N=*68) | | curative setting *(N=6)* | |  |
| Number of concordant studies | 53 (66%) | | 52 (70%) | 1 (17%) | |  | | 65 (70%) | | | | 61 (70%) | | 4 (67%) | | |  | | 46 (62%) | | | | 43 (63%) | | 3 (50%) | |  |
| Spearman's rho |  | | 0.50 (*P*=0.010) |  | |  | |  | | | | 0.57 (*P*<0.001) | |  | | |  | |  | | | | 0.48 (*P*<0.001) | |  | |  |
| Cohen's Kappa | 0.33 (*P*=0.003) | | 0.41 (*P*<0.001) |  | |  | | 0.39 (*P*<0.001) | | | | 0.39 (*P*<0.001) | |  | | |  | | 0.24 (*P*=0.040) | | | | 0.26 (*P*=0.037) | |  | |  |
| Abbreviations: ESMO-MCBS v1.1: European Society for Medical Oncology - Magnitude of Clinical Benefit Scale version 1.1; ASCO-VF v2: American Society of Clinical Oncology - Value Framework version 2; OLUtool v2: OLUtool version 2; *P* = p-value; | | | | | | | | | | | | | | | | | | | | | | | | | | |  |
|  |  |  | | |  | | | |  |  | | |  | | |  | | | | |  |  | |  | |  | |
| **Table A.3b. Sensitivity analysis: Concordance and correlation of studies in which the same outcomes were evaluated between the different frameworks with threshold calculated with the ROC-curve** | | | | | | | | | | | | | | | | | | | | | | | | | | |  |
|  | ESMO-MCBS/ASCO-VF v2 | | | | | |  | | | | | | | | | | | | ASCO-VF v2/OLUtool v2 | | | | | | | |  |
|  | all studies (*N*=80) | | palliative setting (*N*=74) |  | |  | |  | | |  | | | |  | | |  | | all studies (*N*=74) | | | palliative setting (*N*=68) | |  | |  |
| Optimal cut-off for ASCO-VF v2 | 46.6 p | | 49.15 p |  | |  | |  | | |  | | | |  | | |  | | 55.65 p | | | 55.65 p | |  | |  |
| Number of concordant studies | 56 (70%) | | 56 (76%) |  | |  | |  | | |  | | | |  | | |  | | 48 (65%) | | | 46 (68%) | |  | |  |
| Spearman's rho |  | | 0.50 (*P*<0.001) |  | |  | |  | | |  | | | |  | | |  | |  | | | 0.47 (*P*<0.001) | |  | |  |
| Cohen's Kappa | 0.40 (*P*<0.001) | | 0.51 (*P*<0.001) |  | |  | |  | | |  | | | |  | | |  | | 0.33 (*P*=0.001) | | | 0.38 (*P*<0.001) | |  | |  |
| Abbreviations: ESMO-MCBS v1.1: European Society for Medical Oncology - Magnitude of Clinical Benefit Scale version 1.1; ASCO-VF v2: American Society of Clinical Oncology - Value Framework version 2; OLUtool v2: OLUtool version 2; p: points; *P* = p-value | | | | | | | | | | | | | | | | | | | | | | | | | | |  |

**Supplementary S3 Table**: Sensitivity analysis for the concordance and the correlation between the studies considering studies with the same evaluated endpoints only.
