# Supplementary material for: Clinical benefit of cancer drugs approved in Switzerland 2010–2019
Source: PLoS One. 2022 Jun 10;17(6):e0268545. doi: 10.1371/journal.pone.0268545 (PMC9187080; doi:10.1371/journal.pone.0268545)
Supplement: S4 Table — (DOCX) [file pone.0268545.s004.docx]

**Supplementary S4 Table**: Predictors of substantial clinical benefit on univariable logistic regression for all studies.

|  |  |  | ESMO-MCBS v1.1 (all studies) | | |  | ASCO-VF v2 (all studies) | |  | OLUtool v2 (all studies) | |
| --- | --- | --- | --- | --- | --- | --- | --- | --- | --- | --- | --- |
| **Univariable analysis** | | | OR (95% CI) | | *P* |  | OR (95% CI) | *P* |  | OR (95% CI) | *P* |
|  | Randomized Patients | | | 1.00 (1.00-1.00) | 0.105 |  | 1.00 (0.99 - 1.00) | 0.195 |  | 1.00 (1.00 - 1.00) | 0.854 |
|  | Analysed Patients | | | 1.00 (1.00 -1.00) | 0.118 |  | 1.00 (0.99 - 1.00) | 0.117 |  | 1.00 (1.00 - 1.00) | 0.983 |
|  | Line of treatment | | |  |  |  |  |  |  |  |  |
|  |  | Neoadjuvant/adjuvant | | **10.91 (1.26 - 94.52)** | **0.030** |  | 0.17 (0.02 - 1.54) | 0.116 |  | 2.44 (0.43 - 13.99) | 0.317 |
|  |  | First line (vs. further line) | | 1.39 (0.59 - 3.26) | 0.450 |  | 1.12 (0.47 - 2.71) | 0.796 |  | **2.09 (0.90 - 4.83)** | **0.087** |
|  | Phase 3 (vs. phase 1, 2) | | | **15.91 (2.03 - 124.86)** | **0.008** |  | 0.68 (0.11 - 4.31) | 0.685 |  | **5.68 (1.52 - 21.16)** | **0.010** |
|  | Blinded study (vs. open label) | | | 0.83 (0.37 - 1.86) | 0.649 |  | **0.46 (0.19 - 1.09)** | **0.078** |  | **3.47 (1.50 - 8.03)** | **0.004** |
|  | Cross-over allowed (versus not) | | | **3.09 (1.05 - 9.09)** | **0.041** |  | 1.78 (0.61 - 5.19) | 0.293 |  | 1.85 (0.63 - 5.41) | 0.263 |
|  | One primary endpoint (versus more than one) | | | 1.24 (0.45 - 3.44) | 0.682 |  | 1.22 (0.44 - 3.37) | 0.708 |  | 0.95 (0.35 - 2.60) | 0.924 |
|  | Primary endpoint overall survival | | | 1.42 (0.63 - 3.20) | 0.403 |  | 0.57 (0.24 - 18.78) | 0.200 |  | 0.54 (0.24 - 1.23) | 0.143 |
|  | HRQoL benefit as secondary outcome (yes vs. no) | | | **8.59 (2.24 - 32.98)** | **0.002** |  | **4.88 (1.27 - 18.78)** | **0.021** |  | 1.92 (0.63 - 5.88) | 0.255 |
|  | Approved since 2017 (vs. 2010 - 2016) | | | 1.73 (0.76 - 3.94) | 0.194 |  | **0.46 (0.20 - 1.10)** | **0.081** |  | 1.81 (0.80 - 4.10) | 0.154 |
|  | Companion diagnostics | | | 1.00 (0.44 - 2.27) | 1.00 |  | 2.14 (0.86 - 5.32) | 0.101 |  | 1.28 (0.57 - 2.88) | 0.551 |
| Abbreviations: ESMO-MCBS v1.1: European Society for Medical Oncology - Magnitude of Clinical Benefit Scale version 1.1; ASCO-VF v2: American Society of Clinical Oncology - Value Framework version 2; OLUtool v2: OLUtool version 2; OR: odds ratio; 95% CI: 95% confidence interval; *P*: p-value; vs.: versus; incl.: inclusive; HRQoL: Health related quality of life | | | | | | | | | | | |
