# Supplementary material for: Clinical benefit of cancer drugs approved in Switzerland 2010–2019
Source: PLoS One. 2022 Jun 10;17(6):e0268545. doi: 10.1371/journal.pone.0268545 (PMC9187080; doi:10.1371/journal.pone.0268545)
Supplement: Attachment — Submitted filename: Respone_to_Reviewers_final.doc [file pone.0268545.s006.doc]

**Re: PONE-D-21-28268**

**Clinical Benefit of cancer drugs approved in Switzerland 2010 - 2019**

**Response to Reviewers**

**Editor:**

1. Please ensure that your manuscript meets PLOS ONE's style requirements, including those for file naming.

*Authors’ reply*

- *We have named the files according to PLOS ONE's style requirements.*
- *We have followed the formatting guidelines of the manuscript.*

2. Thank you for stating the following in the Competing Interests section: “…” Please confirm that this does not alter your adherence to all PLOS ONE policies on sharing data and materials, by including the following statement: ""This does not alter our adherence to PLOS ONE policies on sharing data and materials.” (as detailed online in our guide for authors http://journals.plos.org/plosone/s/competing-interests). If there are restrictions on sharing of data and/or materials, please state these. Please note that we cannot proceed with consideration of your article until this information has been declared.

Please include your updated Competing Interests statement in your cover letter; we will change the online submission form on your behalf.

*Authors’ reply*

- *We have updated the conflict of interest statement and have now stated that these do not alter our adherence to PLOS ONE policies on sharing data and materials.*

3. We noted in your submission details that a portion of your manuscript may have been presented or published elsewhere. [DETAILS AS NEEDED] Please clarify whether this [conference proceeding or publication] was peer-reviewed and formally published. If this work was previously peer-reviewed and published, in the cover letter please provide the reason that this work does not constitute dual publication and should be included in the current manuscript.

*Authors’ reply*

- *The main findings of our work were presented at the annual meeting of the European Society of Medical Oncology (ESMO) in 2020. An abstract of this oral presentation was published: Annals of Oncology (2020) 31 (suppl_4): S903-S913. 10.1016/annonc/annonc287 (297 words plus 1 table). Presentation of an abstract at a conference does not constitute dual publication and we confirm that our work submitted for publication in PLOS ONE has not yet been published elsewhere and is not under consideration for publication elsewhere.*

4. In your Data Availability statement, you have not specified where the minimal data set underlying the results described in your manuscript can be found. PLOS defines a study's minimal data set as the underlying data used to reach the conclusions drawn in the manuscript and any additional data required to replicate the reported study findings in their entirety. All PLOS journals require that the minimal data set be made fully available. For more information about our data policy, please see <http://journals.plos.org/plosone/s/data-availability>.

Upon re-submitting your revised manuscript, please upload your study’s minimal underlying data set as either Supporting Information files or to a stable, public repository and include the relevant URLs, DOIs, or accession numbers within your revised cover letter. For a list of acceptable repositories, please see http://journals.plos.org/plosone/s/data-availability#loc-recommended-repositories. Any potentially identifying patient information must be fully anonymized.

Important: If there are ethical or legal restrictions to sharing your data publicly, please explain these restrictions in detail. Please see our guidelines for more information on what we consider unacceptable restrictions to publicly sharing data: http://journals.plos.org/plosone/s/data-availability#loc-unacceptable-data-access-restrictions. Note that it is not acceptable for the authors to be the sole named individuals responsible for ensuring data access.

We will update your Data Availability statement to reflect the information you provide in your cover letter.

*Authors’ reply*

- *We are happy to provide the full data underlying our analysis and kindly ask PLOS ONE to make the data available with the article.*

**Reviewers' comments:**

1. Is the manuscript technically sound, and do the data support the conclusions?

The manuscript must describe a technically sound piece of scientific research with data that supports the conclusions. Experiments must have been conducted rigorously, with appropriate controls, replication, and sample sizes. The conclusions must be drawn appropriately based on the data presented.

Reviewer #1: Yes

Reviewer #2: Yes

2. Has the statistical analysis been performed appropriately and rigorously?

Reviewer #1: Yes

Reviewer #2: Yes

3. Have the authors made all data underlying the findings in their manuscript fully available?

The PLOS Data policy requires authors to make all data underlying the findings described in their manuscript fully available without restriction, with rare exception (please refer to the Data Availability Statement in the manuscript PDF file). The data should be provided as part of the manuscript or its supporting information, or deposited to a public repository. For example, in addition to summary statistics, the data points behind means, medians and variance measures should be available. If there are restrictions on publicly sharing data—e.g. participant privacy or use of data from a third party—those must be specified.

Reviewer #1: Yes

Reviewer #2: Yes

4. Is the manuscript presented in an intelligible fashion and written in standard English? PLOS ONE does not copyedit accepted manuscripts, so the language in submitted articles must be clear, correct, and unambiguous. Any typographical or grammatical errors should be corrected at revision, so please note any specific errors here.

Reviewer #1: Yes

Reviewer #2: Yes

5. Review Comments to the Author

**Reviewer #1:**

The authors of the mentioned manuscript investigated the clinical benefit of different solid cancer drugs approved in Switzerland as measured by different published scoring systems. The article discusses a relevant topic and is written in an intelligible fashion, the statistics are performed correctly and the results are discussed in a differentiated manner.

*Authors’ reply*

*We thank the reviewer for his/her favorable opinion of our work.*

**Reviewer #2:**

The authors collected over 100 studies of approved cancer drugs and compared 3 different published frameworks that measure the clinical benefit and efficacy of the new treatments. Based on the measures of the frameworks only half of the approved drugs showed a substantial clinical benefit. The study also represents an impressive collection of drug efficacy and outcome measures and is a very valuable contribution.

*Authors’ reply*

*We thank the reviewer for his/her favorable opinion of our work.*

1. The study performs a global assessment of all studies and subsets and would benefit from including some more details which would dramatically improve the reading and understanding of the paper. For example the data collection is unclear, a list/table of the data matrix of those 100 drugs with the described endpoints and the inputs that were used for each of the 3 frameworks, e.g. OS, PFS, DFS, RFS, ORR, toxicity, QoL outcomes and missing data and framework scoring.

*Authors’ reply*

*We fully agree that sharing data underlying research is desirable and have now uploaded the full data underlying our analysis.*

2. Is also unclear which predictors were used and passed statistical significance (section Statistical analysis/ Data Synthesis Scoring).

*Authors’ reply*

*As stated in the methods section only variables with a p-value < 0.1 in the univariable analysis were included in multivariable analyses. We have now also added this information in the footage of Table 3 and have also included a reference to Supplementary Table S4 where the data from univariable analyses are presented.*

3. In addition it would make the paper more interesting if individual examples could be briefly highlighted in the discussion section. For example a treatment with exceptional improvement and one example that does not show a substantial clinical benefit across the three chosen frameworks. This could allow to understand the shortcoming of the frameworks. For example would it be possible to make a venn diagram showing the overlap of studies with clinical benefit and without between the frameworks (e.g. Figure 2a)? (Section Substantial clinical benefit)

*Authors’ reply*

*We prefer not to highlight individual studies. Any such picking would be arbitrary and likely biased and would deviate from the main message of our work which is to point out a general concern with lack of benefit in many rather than individual indications. However, given the availability of the full data and scoring underlying our work, readers can easily identify examples of high and low clinical benefit.*

*We acknowledge the idea of including a Venn diagram to compare the ratings of the different frameworks. However, we feel in depth analysis of reasons for discordance of the frameworks is beyond the scope of this work which is to demonstrate that no clinically relevant benefit as documented in a relevant proportion of approved drugs.*

4. One shortcoming seems to be also that the paper (e.g. the discussion section) does not consider to discuss findings of the literature. Currently only a single citation is given in the discussion section (e.g. include similar studies of other countries, new measures/guidelines, statistical approaches, electronic health records, follow-up etc.).

*Authors’ reply*

*The discussion section contains reference to other analyses in the literature, namely studies supporting cancer drug approvals by the FDA, the EMA and the Canadian Agency for Drugs and Technologies in Health (references 11,12,24). We agree that further research is necessary to better understand the benefits of newly approved drugs in the community and have added a respective statement.*

5. Other comments

Background: check sentence. There are different validated tools ... of a cancer drugs

Discussion: We found that only around half of the included 'trials' showed ...

*Authors’ reply*

*We thank the reviewer for pointing out the two sentences. We have now revised the wording to make the sentences more clear and logical.*
